# Human ex vivo spinal cord slice culture as a useful model of neural development, lesion, and allogeneic neural cell therapy

**DOI:** 10.1186/s13287-020-01771-y

**Published:** 2020-07-29

**Authors:** Chenhong Lin, Cinzia Calzarossa, Teresa Fernandez-Zafra, Jia Liu, Xiaofei Li, Åsa Ekblad-Nordberg, Erika Vazquez-Juarez, Simone Codeluppi, Lena Holmberg, Maria Lindskog, Per Uhlén, Elisabet Åkesson

**Affiliations:** 1 Department of Neurobiology, Care Sciences and Society, Div. of Neurogeriatrics, Karolinska Institutet, Stockholm, Sweden; 2 Department of Neurology and Laboratory of Neuroscience, Università degli Studi diMilan, Milan, Italy; 3 Division of Molecular Neurobiology, Departmentof Medical Biochemistry and Biophysics, Karolinska Institutet, Stockholm, Sweden; 4 Department of Neurology, Shengjing Hospital of China Medical University, Shenyang, People’s Republic of China; 5 Department of Clinical Science, Intervention and Technology, Div. of Obstetrics and Gynecology, Karolinska Institutet, Stockholm, Sweden; 6 The R&D Unit, Stockholms Sjukhem, Stockholm, Sweden

**Keywords:** Spinal cord injury, Stem cell therapy, Human organotypic culture

## Abstract

**Background:**

There are multiple promising treatment strategies for central nervous system trauma and disease. However, to develop clinically potent and safe treatments, models of human-specific conditions are needed to complement in vitro and in vivo animal model-based studies.

**Methods:**

We established human brain stem and spinal cord (cross- and longitudinal sections) organotypic cultures (hOCs) from first trimester tissues after informed consent by donor and ethical approval by the Regional Human Ethics Committee, Stockholm (lately referred to as Swedish Ethical Review Authority), and The National Board of Health and Welfare, Sweden. We evaluated the stability of hOCs with a semi-quantitative hOC score, immunohistochemistry, flow cytometry, Ca^2+^ signaling, and electrophysiological analysis. We also applied experimental allogeneic human neural cell therapy after injury in the ex vivo spinal cord slices.

**Results:**

The spinal cord hOCs presented relatively stable features during 7–21 days in vitro (DIV) (except a slightly increased cell proliferation and activated glial response). After contusion injury performed at 7 DIV, a significant reduction of the hOC score, increase of the activated caspase-3^+^ cell population, and activated microglial populations at 14 days postinjury compared to sham controls were observed. Such elevation in the activated caspase-3^+^ population and activated microglial population was not observed after allogeneic human neural cell therapy.

**Conclusions:**

We conclude that human spinal cord slice cultures have potential for future structural and functional studies of human spinal cord development, injury, and treatment strategies.

## Background

Human central nervous system (CNS) lesions, such as spinal cord injury (SCI), present limited spontaneous regenerative properties and largely lack available treatments offering functional improvement. Numerous and often promising pre-clinical studies with various neuroprotective compounds, factors supporting regeneration as well as cell therapies have led to multiple clinical trials [1–4]*.* However, translation from experimental animal models to the clinical settings has proven to be challenging.

State-of-the-art treatment for traumatic SCI aims to limit the lesion to a minimum but neural regeneration and functional recovery after SCI is still limited. Neural cell therapy represents one experimental treatment strategy to support structural and functional improvement after SCI with promising and significant results in SCI animal models [5–9]. Recently, human allogeneic neural cell therapy clinical trials in chronic SCI [10, 11] presented feasibility and tolerability. However, statistical power and control groups are missing often due to ethical reasons. Therefore, to improve treatment efficacy and ensure safety, we need model systems mimicking human nervous system lesions and allogeneic conditions as closely as possible to elucidate mechanisms involved in injury progression as well as neuroprotection and repair.

In vivo animal models permit the study of complex interactions between multiple organ systems. However, using animal studies alone is not sufficient to understand human biology and pathology. Compared to regular cell culture, ex vivo tissue slice culture provides at least partly maintained original tissue architecture with some intact functional neural networks [12]. Ex vivo slice cultures are relatively easy to establish and can be utilized in short-(days) as well as long-(weeks) term experiments. Moreover, they may recapitulate or mimic lesion pathology, allowing the study of pathological and regenerative pathways in tissue-like complexity [13, 14]. Therefore, an established human nervous system slice culture model would allow promising repair strategies including gene [15, 16] as well as neural cell therapeutic approaches [17–20] to be pre-clinically tested in human conditions. Thus, as a complementary method (allowing human spinal cord study, while reducing costs and resource demands in addition to animal use), human slice culture, when available, is of value.

So far, CNS slice culture with tissue from embryonic, postnatal, and adult stages has mainly been utilized in rodents. Both cross-sectional and longitudinal organotypic slice cultures from animal spinal cord have been developed [21–26] and used to study SCI [27–29] and cell therapy [17–20]. However, to develop treatment strategies for human SCI, studies of species-specific anatomical, physiological, and immunological features [30–32] are central [33, 34] and should be further utilized*.*

In this paper, we have developed a human ex vivo spinal cord slice culture model and described a method to evaluate such cultures in vitro. With the present protocol, we report longitudinal and cross-sectional human tissue slice cultures of the spinal cord with markedly consistent organotypic structural features after 7–21 days in vitro (DIV). We also confirmed their viability by Ca^2+^ signaling and electrophysiological analysis. To further use the model, we performed in vitro slice contusion injury and studied allogeneic neural cell therapy by grafting human first trimester-derived neural stem/progenitor cells (hfNPCs), to lesioned or control slices. We conclude that human spinal cord tissue slice culture is a suitable complementary platform to study human spinal cord development, injury, and treatment strategies.

## Methods

### Human tissue collection

Human first trimester embryonic/fetal brain stem and spinal cord tissues (5–10.5 weeks postconception (w.)) were collected after elective abortions with maternal oral and written informed consent and following ethical permission from the Regional Human Ethics Committee, Stockholm (lately referred to as Swedish Ethical Review Authority), and the National Board of Health and Welfare, Sweden. The postconceptional age of tissue was determined by examination of anatomical landmarks according to the atlas of normal human development [35]. The tissues were dissected in physiological sodium chloride solution under sterile conditions. Directly thereafter, tissues were cut with a McIlwain chopper and processed as described below.

### Experimental design

**Fig. 1:**
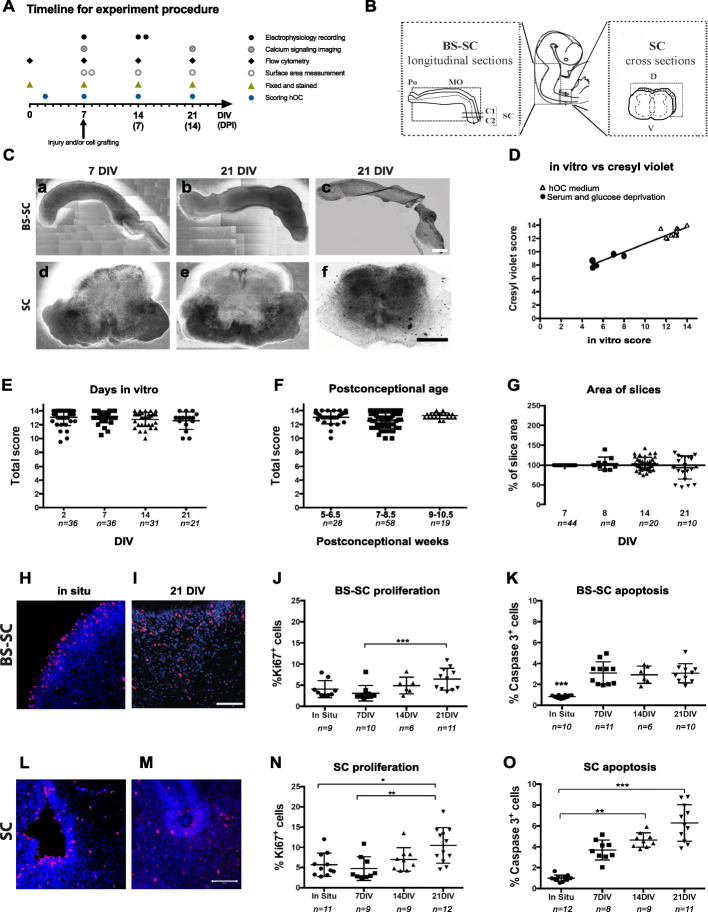
Timeline and quality of slices during culture. **A** Schematic timeline for the experimental procedure. **B** Schematic picture of the longitudinal BS-SC and cross-sectional SC slice cultures. **C** Appearance of longitudinal BS-SC and cross SC sectional slices at 7 (a, d) and 21 (b, e) DIV. In (c) and (f), Cresyl violet staining at 21 DIV of BS-SC and SC slice cultures, respectively, are shown. **D** The correlation between the total in vitro hOC score and total postfixation Cresyl violet-based hOC score (*n* = 16 slices from 3 biological cases) is presented. The two total scores were correlated (*r*_*s*_ = 0.8440) and linearly distributed (*r*^2^ = 0.9353; *p* < 0.0001). **E** Total hOC scores of slices cultured under normal conditions at 2, 7, 14, and 21 DIV. **F** Total hOC score of samples grouped on the basis of their postconceptional week. **G** The slice area over time in in vitro culture. The area at 7 DIV was considered as 100%. **H**, **I** Ki-67 expression in BS-SC slices in in situ and at 21 DIV, respectively. **J**, **K** Flow cytometry quantification of proliferating (**J**) and apoptotic (**K**) cells in BS-SC slices. **L**, **M** Immunostaining for Ki-67 on SC slices in situ (**L**) and 21 DIV (**M**). **N**, **O** Flow cytometric quantification of proliferating (**N**) and apoptotic (**O**) cells in SC slices. The flow cytometry data is presented as the percentage of positive cells out of total cell number. Values are presented as mean ± SEM. Bars: a − c = 2 mm; d − f = 0.5 mm. **H**, **I**, **L**, **M** = 0.1 mm. *p < 0.05; **p < 0.01; ***p < 0.001; ****p < 0.0001. Abbreviations: BS-SC, longitudinal brain stem-spinal cord slices; SC, spinal cord cross-sectional slices; DIV, days in vitro; *n*, number of slices (**E–G**) or biological cases (**J**, **K**, **N**, **O**)

Human spinal cord tissue was collected and ex vivo cross- or longitudinal sections processed for slice culture (in the following text referred to as human organotypic slice culture, hOC) on day 0. In order to validate our ex vivo slice culture scoring system, we scored the slices in vitro during culture by phase contrast microscopy at 2 DIV, 7 DIV, 14 DIV, and 21 DIV as well as fixed slices at 0 (in situ), 7 DIV, 14 DIV, and 21 DIV, respectively, for histo- and immunohistochemical analysis for comparison, as illustrated in Fig. 1A. We also measured the area of each slice at 7–8 DIV, 14 DIV, and 21 DIV. In order to validate the functionality of the hOC slices, we imaged and measured Ca^2+^ signaling at 14 and 21 DIV and performed electrophysiological recordings at 7 and 14–15 DIV. With the aim to evaluate acute effects of allogeneic experimental hfNPC therapy in “SCI,” we performed slice contusion at 7 DIV, and 1 h afterwards, sham or hfNPC grafting was applied in lesioned or control slices. Slices with and without lesions and grafts were analyzed by immunohistochemistry as well as flow cytometry. For an overview of the design of study, see Fig. 1A.

### hfNPC culture

hfNPCs were initially isolated from human first trimester spinal cord (5–7.5 w.) after consent procedures as described above. The tissues were mechanically homogenized with a Teflon glass homogenizer and cultured as neurospheres as previously described by us [36–40]. A vast majority of the hfNPCs were nestin-positive neural progenitor cells with only about 0.5% of the cells being Tra-1-60, Tra-1-81, or SSEA-4-positive (all early stem cell markers), with a potential to differentiate into β-tubulin III- (neuronal marker) and GFAP (glial marker)-immunoreactive cells as well as a sparse number of Gal C- and O4-positive (oligodendrocyte markers) cells, with low immunogenic potential [36–40].

The cultures were expanded in the absence of antibiotics up to 7 passages. Mycoplasma tests were performed along the course of culture, and no mycoplasma infected cultures were included in the presented data. Tissues were maintained at 37 °C, 5% CO_2_, and 95% humidity and the medium was changed three times per week.

### Human organotypic culture (hOC)

The culture method applied here is a modification of the interface method described by Stoppini et al. [13]. Human brain stem-spinal cord (BS-SC) longitudinal slices included tissues rostrally from the pontine (PO) flexure and all the way caudally to the second or third cervical segment, (C2–3), while human spinal cord (SC) cross-sections included thoracic or lumbar segments (Fig. 1B). The human tissues were cut with a McIlwain chopper (Tissue Chopper, Ted Pella, Inc., USA) at 300 μm of thickness for longitudinal BS-SC slices or 200 μm of thickness for SC cross-sectional slices and thereafter transferred to culture dishes with DMEM medium.

The slices were placed in 6-well plates on a semi-porous membrane (pore size 0.4 μm, Millipore) pre-treated with poly-D-Lysine and with 1.2 ml of culture medium (DMEM/F12 (1:1) supplemented with glutamax, 25% inactivated horse serum, 0.65% glucose, 15 mM Hepes, 1% penicilin/streptomycin) in each well with the aim to neither cover nor dehydrate the slice [13]. Tissues were maintained at 37 °C, 5% CO_2_, and 95% humidity and the medium was changed two to three times per week.

Some control slices were cultured under serum- and glucose-deprivation (DMEM/F12 (1:1) supplemented with glutamax, 15 mM Hepes, 1% penicillin/streptomycin) when validating our in vitro hOC score as described below.

### In vitro evaluation of slices: hOC score

At 2, 7, 14, and 21 DIV, slice culture appearance was rated under a light inverted phase contrast microscope utilizing a 2.5x objective. Different parameters of the cross-sectional tissue slices were evaluated, such as the maintenance of regional shape, regional landmarks, tissue slice edge integrity, and others described in Table 1, where each parameter was scored on a scale between 0 and 2. The hOC score protocol was developed for SC cross-sectional slices. The area (*A*) of the cross-sectional slices was calculated, *A* = π*r*^2^, where *r* is the average of the dorso-ventral and the lateral-lateral radius in millimeters.

**Table 1:** hOC Score Protocol for Human Spinal Cord (SC) Cross Sections

**Score and parameters** (the total score is the sum of the respective observed scores, max score 14)	
**Regional shape of slice (observed shape of tissue slice compared to an in situ SC cross section)**	
2. Maintained	
1. Partly maintained	
0. Not maintained	
**Anatomical organization of slice (Observed presence of the following listed land marks)**	
*Landmarks:****A)****gray/white matter,****B)****dorsal septum and/or dorsal funiculi,****C)****(incipient) ventral median fissure and/or ventral funiculi,****D)****dorsal horn,****E)****ventral horn,****F)****central canal/neuroepithelial cell layer/ extra canalicular structures*	
*Organotypic landmarks*	
2. ≥ 3 landmarks present, intact and bilateral	
1. ≥ 3 landmarks present but not all intact and/or bilateral	
0. < 3 landmarks present or none of the landmarks are intact, bilateral	
**Edges**	
2. Sharp/even (>75% of total slice edge)	
1. Partly sharp/even (25-75% of total slice edge)	
0. Not sharp/even (sharp edges observed in <25% of total slice edge)	
**“Dark aggregates/dark spots” in tissue slice**	
2. No/few spots	
1. Many (covering ≤50% of slice area (1a scattered/1b aggregates))	
0. Vast number (covering >50% of slice area (0a scattered/0b aggregates))	
**Dispersed cells** (cells observed outside edge of tissue slice)	
2. No or few (observed outside <5% of slice edge)	
1. Many (observed outside 5-50% of slice edge)	
0. Vast number (observed outside >50% of slice edge)	
**Thickness of slice, estimated**	
2. Maintained (within >75% of total area)	
1. Partly maintained (within 25-75% of total area)	
0. Not maintained (thickness maintained within <25% of total area)	
**Tissue integrity**	
2. Intact tissue structural integrity (within >75% of total slice area)	
1. Partly intact tissue structural integrity (within 25-75% of total slice area)	
0. Not intact tissue structural integrity (structural integrity maintained within <25% of total slice area)	
**TOTAL SCORE:**	
***Extra observations***	
***Fibers***	
*2. Multiple observed elongated fibers/fiber network (>5 visible outgrowing fibers)*	
*1. One/few (≤5) observed long or short fibers*	
*0. No outgrowing fibers are observed*	
***Size (mm)***	
*Length of slice along dorso-ventral axis*	
*Length of slice along lateral-lateral axis*	

Same slices were evaluated and scored by three independent observers, first during in vitro culture under phase contrast microscope at 7–8 DIV and 14–15 DIV, and then second after fixation and histochemistry by light microscopy. The intra- and inter-rater variability of the hOC score was calculated. The slices that at 7 DIV had a total score lower than 10 out of maximum 14 points, and thereby presented lost anatomical orientation and/or structural integrity, were excluded from experimental contusion lesion and human allogeneic cell therapy studies.

### Immunohistochemistry and histochemistry

At 0 (in situ tissue slices, not cultured), 7, 14, and 21 DIV, hOC slices were fixed in 4% paraformaldehyde (PFA) diluted in 0.01 M phosphate-buffered saline (PBS) for 2 h at 4 °C. Thereafter, hOCs were processed for whole mount staining, or for staining of individually cryostat-cut sections, 10 μm.

During both procedures, the specimens were washed by Tris buffer saline × 1 with 0.005% Tween-100× and blocked in blocking solution (0.01 M PBS, 0.1% Triton X-100, 4% bovine serum albumin and 2% normal goat serum). The primary and secondary antibodies (Suppl. Table 1) were also diluted in the blocking solution. Hoechst 33342 (1:400) was applied as a final step used to label cell nuclei prior to the mounting of glass cover slips with poly vinyl-alcohol (0.1 mg/ml) in DABCO (1,4-diazabicyclo [2.2.2] octane, 0.03 mg/ml). Images were acquired with a confocal microscope utilizing the 20x or 40x objectives and ZEN 2009 software.

Free-floating tissue slice immunohistochemistry was performed on hOC slices while they were still attached to the culture membrane. All incubations were done at room temperature (RT) and with mild shaking. After a rinse in washing buffer, slices were incubated in blocking solution for 1 h, followed by an overnight incubation with the primary antibodies. After incubation with secondary antibodies (2 h in the dark), specimens were stained with Hoechst and mounted on glass slides.

BS-SC and SC slices were cryopreserved in 30% sucrose in 0.01 M PBS over night at 4 °C, gently removed from the membrane, transferred to cryomolds, embedded in Tissue Tek –OCT compound, and left over night at 4 °C. The in situ control (non-cultured) BS-SC and SC slices were cryopreserved for 24 h in 30% sucrose at 4 °C. Thereafter, the samples were frozen in 2-Methylbutan and stored at − 20 °C until sectioned at 10 μm thickness with a cryostat. Then the cryostat cut sections were heated at 95–100 °C for 20 min in sodium citrate buffer (10 mM). Specimens were blocked in blocking solution 1 h as described above, followed by incubation in primary antibodies, overnight at 4 °C. After rinses, secondary antibodies were added for 2 h at RT, followed by Hoechst labeling of cell nuclei also as described above.

Cresyl violet staining was performed on cryostat cut sections or whole mount slices. Slices and sections were evaluated at 0 (in situ control slices), 7, 14, and 21 DIV and stained and scored with the same hOC score protocol developed for in vitro analysis during culture (Table 1).

Quantification based on immunohistochemistry was conducted with images that were randomly taken in each condition. DAPI^+^ cells were counted automatically by ImageJ, with the same filter for all sections. Marker and DAPI double-positive cells were considered as marker-positive cells.

### Flow cytometry

BS-SC and SC slices were analyzed by flow cytometry to quantify the percentages of proliferative, apoptotic, glial, and immune cells out of the total tissue slice cell number in normal, injured, and/or grafted hOC groups.

Three organotypic slices from each biological case were pooled to run the analysis. After two washes in PBS, slices were digested in TrypLE with 0.1% DNase I at 37 °C for 30 min, and mechanically dissociated into single-cell suspensions. Cells were immunolabeled with the respective antibodies (Suppl. Table. 2) at 4 °C for 30 min and washed and fixed in Cytofix fixation buffer (BD Biosciences). Cytofix/Cytoperm Fixation/Permeabilization Kit (BD Biosciences) were used for intracellular staining following the manufacturer’s instructions. The stained cells were analyzed by flow cytometry with a FACScalibur (Becton and Dickinson) and the percentage of positive cells out of total number of hOC cells analyzed by the software FlowJo (Tree Star, Inc.).

### Calcium signaling

SC hOCs maintained in vitro for 7 or 21 days were bulk- and bath-loaded in culture media simultaneously with Oregon Green BAPTA-1 AM (OGBP1-AM) in Pluronic F-127 containing 20% DMSO. OGBP1-AM (40 mM) of 2 μl was added on the surface of the slice and additional OGBP1-AM was added to the medium at a final concentration of 40 μM. OGBP1-AM-loaded slices were then incubated for 30 min at 37 °C, 5% CO_2_. The slices were then rinsed twice in KREBS-Ringer’s solution (119 mM NaCl, 2.5 mM KCl, 1 mM NaH_2_PO_4_, 2.5 mM CaCl_2_, 1.3 mM MgCl_2_, 20 mM HEPES, and 11 mM glucose). Before imaging, a drop of BD Matrigel™ (BD Biosciences) was added on top of the slices to minimize movement artifacts. The Matrigel was left to solidify by incubating the slices 2 min at 37 °C. Slices on the culture membranes were imaged in KREBS-Ringer’s solution at RT and stimulated with 100 μM ATP. Images were acquired at 0.5 Hz using a Zeiss LSM510 META NLO 2-photon laser scanning microscope equipped with a Ti:Sapphire Chameleon Ultra2 laser (Coherent) tuned at 810 nM using a × 40/0.8 water immersion objective (Zeiss).

Cells were manually selected using Fiji [41] and the Ca^2+^ response was quantified with a custom Python script. For each cell, the baseline signal (F0) was calculated by averaging the integrated intensity measured before ATP stimulation. The Ca^2+^ response is represented by the ratio between the integrated signal intensity at a specific time point and the baseline. A cell was classified as responder if a peak in signal intensity was above 10% of the baseline.

### Electrophysiology

Longitudinal and cross-sectional hOCs were transferred to a submerged recording chamber and constantly perfused (3 ml/min) with oxygenated standard artificial cerebrospinal fluid (aCSF) containing (in mM) 130 NaCl, 3.5 KCl, 1.25 NaH_2_PO_4_, 24 NaHCO_3_, 2 CaCl_2_, 1 MgCl_2_, and 10 glucose with pH 7.4. Extracellular recording pipettes were made from borosilicate capillaries and filled with standard aCSF. Field potentials were evoked by a single pulse of 50 μs duration delivered at 0.03 Hz, and the intensity was maintained in a range between 200 μA and 2 mA; a stimulus was delivered using a concentric bipolar stimulating electrode (FHC, ME, USA). The distance between the stimulating electrode and the extracellular recording pipette varied between 500 and 800 μm along the rostro-caudal axis in the longitudinal slices and was adjusted to obtain an optimal response clearly separated from the stimulus artifact. Recordings were digitized at 10 kHz, stored on a personal computer, and analyzed off-line using pCLAMP 10.

### Experimental SCI and hfNPC therapy

SCI was performed by utilizing the Horizon Infinity spinal cord impactor (Precision Systems and Instrumentation, LLC, USA) set at 25 kDyn (BS-SC hOC slices, *n* = 26; SC hOC slices, *n* = 223).

First, hfNPCs were labeled overnight by adding CellTracker™ Orange CMTMR (5-(and-6)-(((4-Chloromethyl)Benzoyl)Amino) Tetramethylrhodamine (Life Technologies), at a concentration of 2.5 μM in the medium and then washed with fresh medium for 1 h. Neurospheres with a diameter range within 250–400 μm were collected under a dissection microscope and washed in organotypic culture medium. Thereafter, one to two neurospheres (depending on diameter of neurosphere to graft equivalent cell numbers) were grafted to the injury site (Suppl. Fig. 5A-I) 1 h postlesion. In addition, we tested hfNPC grafting (again with equivalent procedure as for the contusion SCI) after a mechanical cut of the unilateral dorsal horn (immature dorsal alar plate) of SC cross-sectional slices (Suppl. Fig. 5J-W).

### Statistical analyses

All statistical analyses were performed using Instat3 and Prism6 (GraphPad Software Inc., USA) for Macintosh computers. Correlation and regression analyses for in vitro and Cresyl violet total hOC score were performed using Spearman’s nonparametric correlation (*r*_*s*_) followed by regression (*r*^2^) analysis. Analysis of possible differences among groups from different postconceptional ages or the groups evaluating neural cell therapy study were performed by the nonparametric Kruskal-Wallis test followed by Dunn’s post hoc test. For the Ca^2+^ signaling data, two-tailored statistical analyses were performed using Mann-Whitney’s nonparametric correlation. *P* values less than 0.05 were considered statistically significant. Results are presented as mean ± SEM. The number (*n*) of biological cases and or slices used in each study is indicated in corresponding results and figures.

## Results

First, we established BS-SC and SC hOCs derived from 5 to 10.5 w. while evaluating stability, tissue organization, cellular heterogeneity (Fig. 1), and functional viability up to 21 DIV. Then, we applied and tested the hOCs as a human ex vivo SCI model and assessed experimental human allogeneic neural cell therapy.

### Generation of hOC slices from first trimester human CNS

#### In vitro observations by phase contrast microscopy

To assess hOC stability and quality, we examined slices under a phase contrast microscope daily up to 21 DIV (Fig. 1C). Both BS-SC and SC hOC slices were relatively well maintained regarding their regional tissue slice shape, edges, anatomical landmarks, and tissue integrity up to 21 DIV (Fig. 1D) with postconceptional ages ranging between 5 and 10.5 w. However, there were some exceptions in BS-SC slices, where the tissue integrity of the BS region in older cases (9–10.5 w. group) at 14 DIV and 21 DIV was compromised, slice area increased, gray and white matter areas were difficult to distinguish, edges were uneven, and dispersed cells covered almost all of the BS perimeter (Suppl. Fig. 1). In SC hOCs, the anatomical organization was stage-dependent with postconceptional age of the tissue. Slices derived from 5 to 6.5 w. naturally presented no (5.5 w.; Suppl. Fig. 1D) or few visible (6–6.5 w.; Suppl. Fig. 1E) landmarks such as dorsal/ventral horns and incipient ventral fissure not yet fully developed at initiation of culture, while slices ≥ 7 w. (Suppl. Fig. 1 A, C) presented a more developed anatomical organization which was preserved up to 21 DIV.

In almost all hOC slices and cases, after 7 DIV or 14 DIV, neuritic fibers were extending in a network around and away from the tissue slices (Suppl. Fig. 1). The in vitro observations of hOCs by phase contrast microscopy were supported by Cresyl violet staining (Fig. 1C (c, f)).

To test the validity of the hOC score protocol, some slices were cultured under serum- and glucose-deprived condition, which resulted in fewer recognizable land marks and slices became further flattened with uneven edges, and the so-called “dark spots” disappearing (Suppl. Fig. 1 F-G).

#### hOC score validation

The slice status was semi-quantitatively evaluated by utilizing a hOC score protocol with a score range of 0–14 (Table 1). The median of absolute differences of each parameter in intra- and inter-observer scores was in the majority of cases 0. The variation between different observers was not significantly different (*p* > 0.05). To further support the validity of our protocol, we stained a subset of slices with Cresyl violet (slice number = 7 deriving from 3 biological cases, *n* = 3) to compare the in vitro hOC score of slices cultivated under normal conditions (Fig. 1D, triangle symbols in graph), or in serum- and glucose-deprived conditions (Fig. 1D, black circle symbols in graph). The ex vivo culture and Cresyl violet-based hOC score correlated strongly (*r*^2^ = 0.84) and presented a linear regression (*r*^2^ = 0.9353; *p* < 0.0001, Fig. 1D), supporting the validity of the hOC score protocol. In this subset of slice cultures, the ones cultured with regular hOC media all got a score value ≥ 10, while slices cultured under deprived conditions all got a total score < 10.

#### Quality of hOC relative to postconceptional age and time in culture

The total hOC scores recorded for all control (uninjured) spinal cord slices (at 2, 7, 14, and 21 DIV) were stable and ranged between 2, 7, and 14, up to 21 DIV (Fig. 1E). When hOC slices were analyzed on the basis of their weeks postconception, no significant differences were detected among the three stage groups (5–6.5, 7–8.5, and 9–10.5 w.), even if the older group (9–10.5 w.) appeared more homogeneous (total score range12–14) when compared to the 5–8.5 w. group (total score range 10–14) (Fig. 1F). These observations suggest that the tissue quality of our human slice culture is stable up to 21 DIV, regardless of the initial postconceptional age of the tissue.

At 14 DIV and 21 DIV, the slice area was non-significantly different from that of 7 DIV (Fig. 1G). The mean thickness of SC slices postfixation was approximately 120 μm including approximately 8–10 cell layers at 21 DIV.

#### Proliferation and apoptosis in hOC

The degree of cell proliferation and apoptosis over time in vitro in hOCs were evaluated respectively, by detecting Ki-67^+^ and activated caspase-3^+^ cells via immunohistochemistry and flow cytometry between 0 (in situ), 7, 14, and 21 DIV (Fig. 1H–O and Suppl. Fig. 3).

The Ki-67^+^ cells in in situ sections (from 5.5, 8, and 10.5 w. cases) were mainly localized in the neuroepithelium around the fourth ventricle (BS-SC longitudinal slices, Fig. 1H) or immature central canal (SC slices, Fig. 1L). At 21 DIV in the hOCs, proliferating Ki-67^+^ cells were scattered all over the parenchyma, irrespectively of the developmental stage (Fig. 1I, M).

The number of Ki-67^+^ cells increased over time in culture, as analyzed by flow cytometry. At 21 DIV, the percentage of this proliferating subpopulation was two-fold larger in both BS-SC (6.4 ± 2.6%) and SC (10.5 ± 4.4%,) compared to that at 7 DIV in BS-SC (3.1 ± 1.8%; *p* < 0.001) and SC (4.7 ± 2.9%); *p* < 0.01 (Fig. 1J, N). When hOCs were compared to in situ slices from the same cases, only SC slices at 21 DIV showed a significant increase of proliferating cells (5*.*7 ± 2.8% and 10.5 ± 4.4%, in situ and 21 DIV, respectively, *p* < 0.05, Fig. 1N). When evaluating whether postconceptional age of original tissues affected proliferation in hOC, the 5–6.5w. group presented a higher percentage of Ki-67^+^ cells at 21 DIV (BS-SC, 9.7 ± 1.1%; SC, 10 ± 2.5%) compared to that at 7 DIV (BS-SC, 2.2 ± 0.4%; SC, 3.8 ± 1.8%) in both BS-SC (*p* < 0.01) and SC slices (*p* < 0.05) (Suppl. Fig. 2A, B). Moreover, at 21 DIV, the BS-SC slices from 5 to 6.5 w. had a significantly higher percentage of proliferating cells compared to the oldest group (10 ± 2.5% and 4.8 ± 2%, respectively; *p* < 0.05) (Suppl. Fig. 2A).

The size of the caspase-3^+^ subpopulation in BS-SC hOCs significantly increased at 7 DIV (3.1 ± 1.1%), 14 DIV (2.9 ± 0.2%), and 21 DIV (3.1 ± 0.9%) (*p* < 0.001, Fig. 1K) compared to in situ (0.8 ± 0.1%) BS-SC slices. In SC slices, caspase-3^+^ cells significantly increased at 14 DIV (4.4 ± 0.1%; *p* < 0.001) and 21 DIV (6.3 ± 1.7%; *p* < 0.05) compared to in situ (0.9 ± 0.3%), while no significant increase was observed at 7 DIV (4.7 ± 2.7%, Fig. 1O).

In BS-SC slices, the 5–6.5 w. group presented a significant elevation (*p* < 0.05) in caspase-3^+^ cells at 14 DIV (3.6% ± 0.7%) and 21 DIV (3.3 ± 1.2%) compared to in situ (0.9 ± 0.1%) (Suppl. Fig. 2C). The apoptosis level was quite stable among the studied postconceptional weeks except for BS-SC at 7 DIV where the older, 9–10.5 w. group showed a significant elevation in caspase-3^+^ cells compared to the youngest group (4.1 ± 0.8% and 2.1 ± 0.2%, respectively; *p* < 0.05, Suppl. Fig. 2C).

The SC hOCs at 21 DIV from the 5–6.5 w. (5.9 ± 2.9%) and 7–8.5 w. (5.8 ± 1.4%) stage groups showed significant increase (*p* < 0.05) in caspase-3^+^ cells compared to the in situ (5–6.5w., 1.1 ± 0.3%; 7–8.5 w.,0.9 ± 0.2%) (Suppl. Fig. 2D), while no significant differences were detected between different postconceptional stages after the same in vitro culture time (Suppl. Fig. 2D).

All together, these results indicate a relatively stable slice viability with less than 8–9% of apoptotic cells in the hOCs up to 21 DIV.

#### Neural cell populations in hOC

Immunohistochemistry was applied to further evaluate the anatomical organization of hOC slices at 21 DIV to that of in situ CNS tissues of 5.5 w., 8 w., and 10.5 w.

**Fig. 2:**
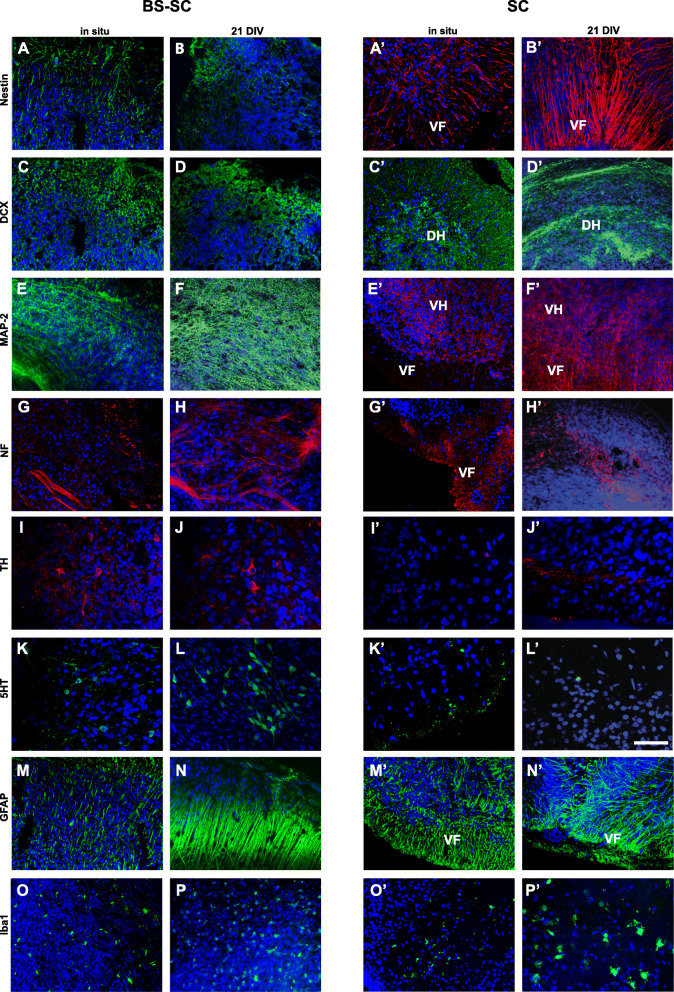
Neural cell populations. Representation of neural populations in BS-SC (**A**–**P**) sections and SC (**A′**–**P′**) whole slices at in situ (from 8.5 to 10.5 w.) and 21 DIV. Expression of nestin (**A,B,A′,B′**), DCX (**C,D,C′,D′** from 5.5 w. case), MAP-2 (**E,F,E′,F′**), NF (**G,H,G′,H′**), TH (**I**, **J**, **I′**, **J′**), 5-HT (**K,L,K′,L′**), GFAP (**M, N, M′, N′**), Iba1 (**O,P,O′P′**). Hoechst (blue) dye was used to stain cell nuclei. Bar: 50 μm in **I**–**L** and **I′**–**L′**; 100 μm in the others. **A**–**M**, **O**–**A′**, **C′**, **E′**, **G′**, **I′**, **K′**, **M′**, **O′**: confocal micrograph from 10 μm section; **N**, **B′**, **D′**, **F′**, **H′**, **J′**, **L’**, **N′**, **P′**: confocal micrograph from whole slice. Abbreviations: BS-SC, longitudinal brain stem-spinal cord slices; SC, spinal cord cross-sectional slices; VF, ventral funiculi; DH, dorsal horns; VH, ventral horns

After 21 DIV, all BS-SC and SC hOCs irrespective of original postconceptional age presented morphological features similar to in situ tissues from 5.5 w. to 10.5 w. (Fig. 2).

In both longitudinal in situ tissues (5.5–10.5 w.) and BS-SC slices at 21 DIV, nestin, DCX, and MAP-2 were abundant in most of the parenchyma (Fig. 2A–F). NF^+^ fibers were mainly organized in bundles (Fig. 2G, H) while TH^+^ and 5-HT^+^ immature cell bodies were grouped in clusters in the brain stem nuclei (Fig. 2I–L), and their immunoreactive fibers ran in the white matter tracts of the human cervical spinal cord. In the SC in situ tissues studied from 5.5 to 10.5 w. as well as in slices at 21 DIV, nestin^+^ cell bodies and fibers were abundantly distributed and aligned all through the tissue slices in both gray and white matter areas (Fig. 2A′, B′).

However, a few differences in cellular distribution were still detected between the hOCs and in situ tissues of respective postconceptional age. At 21 DIV, BS-SC hOCs presented an increase in MAP-2 and GFAP immunoreactivity (Fig. 2F, N), with hypertrophic GFAP^+^ cells as compared to in situ slices (Fig. 2E, M). In in situ tissues (from 5.5 w. to 10 w.), MAP-2^+^ cells were mainly confined to gray matter areas (Fig. 2E′) with fiber bundles running through the white matter, while in hOCs at 21 DIV, MAP-2 was abundant in the whole slice, including the axotomized white matter rim (Fig. 2E′, F′). In in situ tissues, NF^+^ fibers were abundantly detected in the developing white and gray matter, while after 21 DIV, NF^+^ fibers were mainly observed within the gray matter (Fig. 2G′, H′) most probably due to axotomy during slice preparation. At 21 DIV, sparse TH^+^ fibers could be observed in the ventral SC white matter (Fig. 2I′, J′). Fine 5-HT^+^ fibers were also discernible along the white matter in in situ tissues (Fig. 2K′, L′). Iba1^+^ cells in in situ (from 5.5 w. to 10.5 w.) tissues were found sparse but evenly distributed in the tissues (Fig. 2O, O′), while after 21 DIV, Iba1^+^ cells in the SC slices were large and often distributed on the tissue slice surfaces where the tissue had been exposed to the air–liquid interface (Fig. 2P′, air interface side visualized). In in situ SC (5.5–6 w.), DCX was expressed all over the parenchyma, while at 21 DIV, DCX^+^ cells were mainly grouped in clusters in the dorsal horns and along the dorsal white matter (Fig. 2C′, D′).

**Fig. 3:**
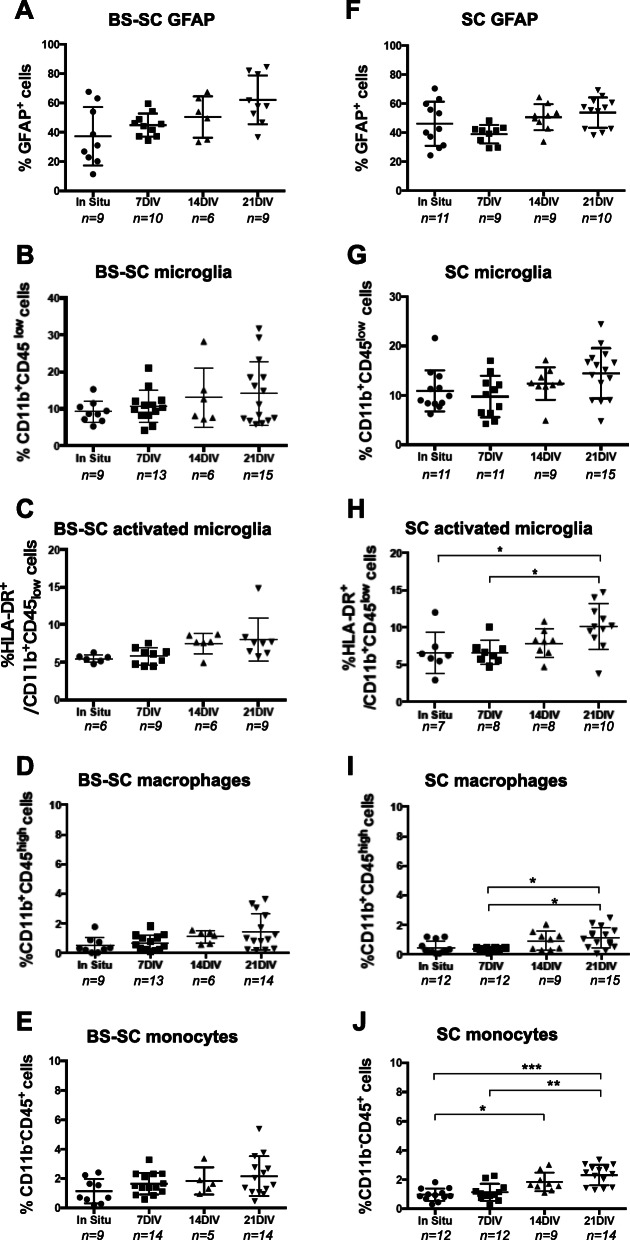
Quantification of glial cells, macrophages and monocytes by flow cytometry. The expression of GFAP (**A**, **F**), CD11b^+^CD45_low_ (**B**, **G**), HLA-DR^+^CD11b^+^CD45_low_ (**C**, **H**), CD11b^+^CD45^high^ (**D**, **I**), and CD11b^−^CD45^+^ (**E**, **J**) was assessed by flow cytometry on slices from in situ and 7, 14, and 21 DIV. The degree of positive cells is presented as percentage of immunoreactive cells out of total cell number. Values are presented as mean ± SEM. *p < 0.05; **p < 0.01; ***p < 0,001. Abbreviations: BS-SC, longitudinal brain stem-spinal cord slices; SC, spinal cord cross-sectional slices; DIV, days in vitro; *n*, number of biological cases analyzed per time point

In addition, glial cell populations were quantified by flow cytometry as GFAP^+^, CD11b^+^ CD45_low_, and HLA-DR^+^/CD11b^+^ CD45_low_ cells (Fig. 3). In both BS-SC and SC slices, the number of GFAP^+^ cells (Fig. 3A, F) and CD11b^+^CD45_low_ cells (total microglia; Fig. 3B, G) did not present a statistically significant change over time in culture. In BS-SC slices, the HLA-DR^+^/CD11b^+^CD45_low_ subpopulation (activated microglia; Fig. 3C) was also observed unaffected over time in culture, while in SC slices at 21 DIV, the percentage of HLA-DR^+^/CD11b^+^CD45_low_ cells (10.1 ± 0.3%) was significantly higher compared to that at 7 DIV (6.6 ± 1.6%) and in situ (6.6 ± 2.7%) *p* < 0.05, Fig. 3H. For representative flow cytometric dot plot graphs, see Suppl. Fig. 4D-F.

To further evaluate the degree of inflammation in our human slice culture, we performed immunohistochemistry with the proinflammatory marker HLA-DR on slice cultures at 7DIV and 14DIV, respectively. We observed low numbers of HLA-DR^+^ cells at these in vitro stages, with no statistically significant difference (0.8% at 7 DIV and 0.7% at 14 DIV) (Suppl. Fig. 4A-C). Altogether, this suggests that the human slice culture model presents (based on the studied markers) relatively stable neuronal subpopulations as well as the degree of inflammation over time in culture, offering opportunity for in vitro human “SCI” study.

#### Cells of hematopoietic origin

Despite all the rinsing during the dissection procedures, a small population of hematopoietic-derived cells was observed in the hOC slices. The developmental first trimester stage studied is also the time when microglial cells deriving from the hematopoietic lineage first appear in the central nervous system. As observed by flow cytometry, 0.5 ± 0.5% of CD11b^+^CD45^high^ (macrophages) and 1 ± 0.4% of CD11b^−^CD45^+^ (monocytes) were present in the tissue samples (Fig. 3D–J). During culture, despite the increase of both populations in SC slices, the mean population sizes still always represented less than 3% of the total cell population. By immunohistochemistry, blood vessels were recognized in in situ tissues, but disappeared over time in culture and no laminin signal was detected at 21 DIV in either BS-SC or SC slice cultures (not shown).

#### Calcium signaling

**Fig. 4:**
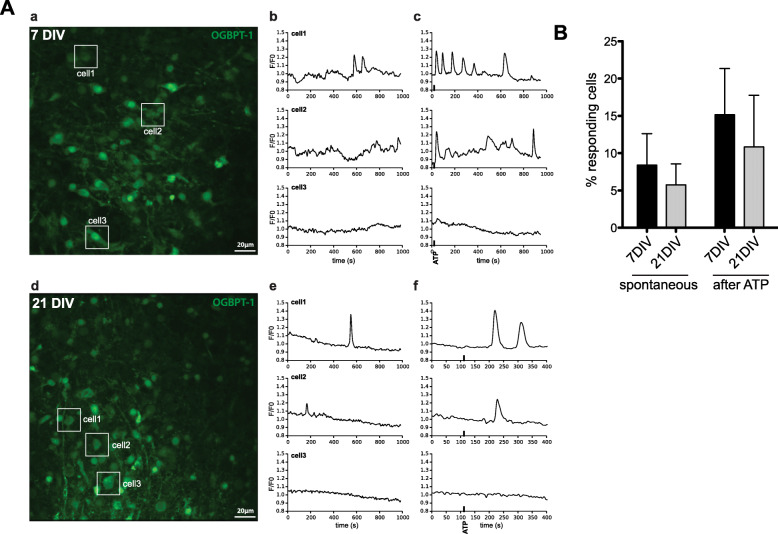
Calcium signaling **A** (a) Representative image of a SC hOC slice (7 DIV) loaded with the Ca^2+^ indicator OGBPT-1. (b) Ca^2+^ traces of three individual cells (shown in a) under basal conditions (before stimulation). (c) Ca^2+^ traces of the same three cells after stimulation with 100 μM ATP (black thick line). (d) Representative image of a SC hOC slice (21 DIV) loaded with the Ca^2+^ indicator OGBT-1. (e) Ca^2+^ traces of three individual cells (shown in d) under basal conditions (before stimulation). (f) Ca^2+^ traces of the same three cells after stimulation with 100 μM ATP (black thick line). **B** Graph showing the percentage of cells with spontaneous Ca^2+^ activity and evoked Ca^2+^ responses after ATP stimulation in SC hOC slices at 7 DIV and 21 DIV. Values are expressed as mean + SEM. Abbreviations: DIV, days in vitro; OGBPT-1, Oregon Green Bapta-1

To determine if the cells from the SC hOCs were functionally active, we assessed spontaneous Ca^2+^ activity at 7 DIV and 21 DIV. Spontaneous Ca^2+^ activity was observed in the cell soma and cell processes both at 7 DIV and 21 DIV (Fig. 4A and Suppl. Video). Noteworthy, the time in culture did not affect the number of responding cells (7 DIV, 8.4 ± 4.2%; 21 DIV, 5.8 ± 2.8%; *p* = 0.77; Fig. 4B), *n* = 4 slices per condition). ATP is an important regulator of intracellular communication in many pathological conditions such as SCI. Therefore, we tested the ability of these first trimester-derived human neural cells to respond to ATP stimulation. We observed that 100 μM ATP was able to elicit cellular Ca^2+^ responses ranging from single transients to oscillatory responses (Fig. 4A, B) without affecting the number of responding cells (7 DIV 15.2 ± 6.2%, 21DIV 10.8 ± 6.9%, *n* = 4).

**Additional file 1: Supplementary video.mp4***:* This is a 20-second video of the calsium imaging experiment, where at the 12th second, ATP was applied.

#### Electrophysiological analysis

**Fig. 5:**
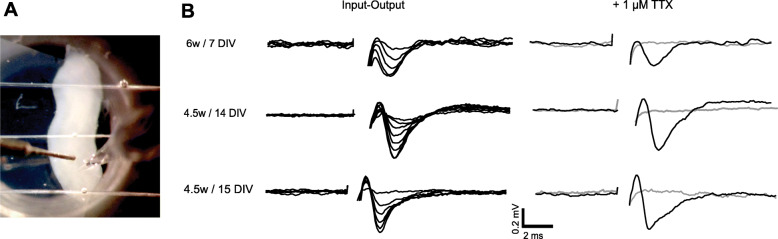
Electrophysiological recordings: Representative stimulus-evoked potentials obtained in longitudinal hOCs derived from 4.5 and 6 w cases and recorded after 7, 14, and 15 DIV. **A** The stimulation electrode and the extracellular recording pipette were placed at distances between 500 and 800 μm along the rostro-caudal axis. Potentials were evoked by single pulse stimulation (50 μs; 0.03 Hz). **B** Input-output traces in the left panel were obtained by increasing the stimulation strength from 200 μA to 2 mA. Preventing neuronal depolarization with 1 μM TTX abolishes stimulus-evoked potentials (gray traces), note that after 30 min wash-out, responses were completely restored (superimposed black trace). Abbreviations: DIV, days in vitro; TTX, tetrodotoxin

To confirm hOC functionality and their ability to transmit neuronal signals, the response to electrical stimulation was assessed in five longitudinal cultures derived from 4.5 w. (two cases) and 6 w., between 7 and 15 DIV. For this purpose, a recording pipette was placed at a distance between 500 and 800 μm from a stimulation electrode along the rostro-caudal axis. Stimulus-evoked potentials were found in all the hOCs tested. Representative superimposed traces, collected after increasing stimulation strength, are shown in Fig. 5; threshold responses were evoked using an intensity of stimulation between 200 and 500 μA and maximal responses ranging between 0.5 and 1 mV were obtained with the stimulation set at 1 to 2 mA. In order to fully confirm the neuronal source of the recorded events, action potentials were blocked using the voltage-gated Na^+^ channel inhibitor, tetrodotoxin (TTX; 1 μM). When present, TTX reversely abolished the stimulus-evoked potentials (Fig. 5).

### Human SCI models and allogeneic neural cell grafting

To experimentally test and utilize the established hOC cross-sectional slices, we mimicked human SCI in the lab in a subset of slices (6–10.5 w.). In addition, experimental human allogeneic neural cell therapy was evaluated utilizing the hOC model system.

#### Ex vivo model of human SCI

An experimental contusion injury was performed on SC slices at 7–8 DIV. The slices were maintained up to 14 days postinjury (DPI) while evaluated by the hOC score, slice area (both in vitro under phase contrast microscopy) measured, flow cytometry, histochemistry, and immunohistochemistry.

**Fig. 6:**
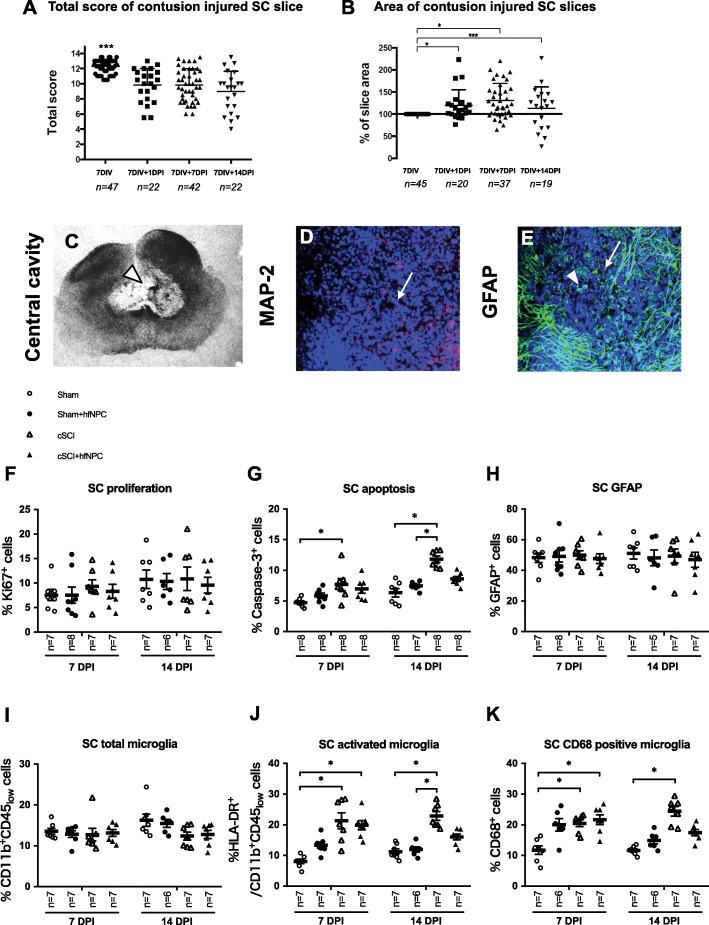
Human SCI model and experimental allogeneic hfNPC grafting. **A** Total hOC Score, and **B** slice area in SC hOCs before (at 7DIV), 1 (7DIV + 1DPI), 7 (7DIV + 7DPI), and 14 (7DIV + 14DPI) DPI; *p < 0.5, ***p < 0.001. **A**, **B***n*, number of slices (from in total 8 biological cases). **C** Appearance of lesion at 14 DPI, phase contrast micrograph with a central cavity, **D** expression of MAP-2 in the center of the lesion, and **E** GFAP immunoreactivity at injury site. Cell nuclei were stained with Hoechst dye. Arrows point out lesion area. **F** Flow cytometer quantification of Ki-67^+^, **G** caspase-3^+^, **H** GFAP^+^, **I** Cd11b^+^CD45_low_, **J** HLA-DR^+^Cd11b^+^CD45_low_, and **K** CD68^+^ cells in sham, sham + hfNPC, contusion “SCI,” and contusion “SCI” + hfNPC at 7 and 14 DPI. The percentage of immunoreactive cells out of total cell number is presented. Values are presented as mean ± SEM. *p < 0.05. Abbreviations: DIV, days in vitro; hfNPC, human fetal neural progenitor cells; DPI, days post injury; *n*, number of biological cases; cSCI, contusion spinal cord injury

Prior to injury, the total hOC score was in all cases above 10 (12.3 ± 0.9), and significantly higher compared to that at 1 DPI (9.8 ± 2.2), 7 DPI (9.8 ± 2), and 14 DPI (9 ± 2.7) (*p* < 0.001). There were no significant differences between the total hOC scores at 1, 7, and 14 DPI (Fig. 6A).

The slice area was unaffected by the contusion injury per se, as observed by phase contract microscope immediately after the contusion lesion. However, the area significantly increased within 24 h after injury (Fig. 6B, *p* < 0.05), even if at 7 and 14 DPI a subpopulation of slices (12% and 35%, at 7 and 14 DPI, respectively) presented a smaller area compared to non-injured slices at 7 DIV (Fig. 6B).

The change of the hOC score reflected that the anatomical organization, tissue integrity, and thickness were altered after experimental contusion SCI. Often, a cavity was observed up to at least 14 DPI (Fig. 6C). Immunohistochemistry revealed markedly reduced MAP-2 immunoreactivity in the lesion area and strong GFAP immunoreactivity surrounding the injury site with several round hypertrophic GFAP^+^ cells present after the lesion (Fig. 6D, E).

#### Allogeneic human neural cell therapy in SCI

Finally, in order to explore the value of the hOCs in testing potential therapeutic strategies, we grafted “donor” allogeneic hfNPC to “host” slices subjected to contusion SCI and compared them to contusion SCI alone or to sham control slices. The donor hfNPCs in the form of neurospheres were placed on top of the “host” human spinal cord slice at time of grafting. The hfNPCs immediately adhered and within just a few hours the neurosphere was integrated into the “host” slice (see Suppl. Fig. 5).

The outcome was evaluated by flow cytometry at 7 DPI and 14 DPI with and without hfNPC grafts.

At 14 DPI, in the injury alone group, both the percentage of active caspase-3^+^ cells, CD68^+^ and HLA-DR^+^/CD11b^+^CD45_low_ cells significantly increased compared to sham (Fig. 6G, J, and K). In the groups where hfNPCs were grafted to slices with a contusion injury, no significant differences were observed at 14 DPI concerning caspase-3^+^ cells and CD68^+^ as well as HLA-DR^+^/CD11b^+^CD45_low_ microglia subpopulations compared to the sham group.

The hfNPCs grafted to “normal” sham slices had no perceptible effect on “host slice tissue” at 7 and 14 DPI compared to sham alone (Fig. 6). Also, no significant difference was observed between 7 DPI and 14 DPI with or without a hfNPC graft just after injury (“SCI” slice contusion) in regard to the percentage of Ki-67, CD11b^+^CD45_low_, or GFAP^+^ cells out of total cell number (Fig. 6F, H, and I).

These findings suggested that hfNPC grafting offers beneficial effects 14 DPI as observed in this human ex vivo slice culture model.

## Discussion

Reliable models to mimic human conditions for study of development, injury mechanisms, and potential therapies in nervous system lesions, such as SCI, are in great demand to complement experimental in vivo, ex vivo, and in vitro animal models, because of structural and functional species differences [30, 31, 42]. Here, we have developed and evaluated a human ex vivo spinal cord slice culture model, allowing us to follow human spinal cord development and SCI pathophysiological mechanisms as well as to test treatment strategies in vitro.

In this paper, the ex vivo human brain stem and spinal cord slice cultures presented remarkably high viability, contained tissue morphology and cellular organization, presence of spontaneous and ATP-induced calcium signaling, and functional neuronal activity in response to electrical stimulation, despite relatively long-term culture up to 21 DIV. We therefore refer to these human ex vivo slice cultures, as organotypic, a term introduced by Reinbold already in 1954 [43, 44].

To our knowledge, there have been only few previously reported attempts to culture human spinal cord tissue in vitro. One ex vivo human spinal cord schwannoma culture [45], one postmortem human spinal cord model derived from adult autopsy tissue [46], and one human fetal spinal cord tissue culture [47]. However, different from these previous reports, the human slice culture method utilized in the present study is an interface-based method (modified from the method by Stoppini et al. [13] and Bonnici and Kapfhammer [48]) that retained relatively intact anatomical structure, local circuitry, glial/neuronal interactions, and viability similar to that reported in cultures of rodent origin [23, 49–51].

In the present paper, a novel and simple hOC score was developed and validated to follow spinal cord cross-sectional slice quality over time in culture and after slice injury. The hOC score was also applied prior to randomly assigning slices either to SCI or control group, to support the selection of slices with equivalent quality. Based on the hOC score, human spinal cord slice culture stability and high replicability were observed up to 21 DIV despite varying original postconceptional stages (5–10.5 w.).

Cell proliferation, as evaluated with the marker Ki-67, was close to double at 21 DIV compared to that in situ and at 7 DIV. With time in culture, a vast spread of Ki-67^+^ cells from the neuroepithelial cell layer to the surrounding developing parenchyma was observed. This may be due to both the denervation at slice preparation and proliferation and cellular migration in the developing spinal cord. Fernandez-Zafra et al., albeit to a lesser degree, reported a similar finding with proliferation in the neuroepithelial cell layers with subsequent migration in adult rodent spinal cord slice cultures [23].

The level of apoptosis, as revealed by the use of marker caspase-3, increased close to 5-fold in the cross-sectional SC slices and 3-fold in the longitudinal BS-SC slice cultures after 7 DIV, as compared to in situ conditions. The apoptosis in the cross-sectional SC slices increased up to 21 DIV. However, the observed apoptotic cell population was not exceeding 7–8% of the total cell number despite the complete rostral and caudal transection at the time of slice preparation depleting supraspinal contact in SC slices. This fact is most probably due to the immature status of the originating human tissue having a high proliferative capacity compensating for the initial cell loss (both by necrosis and apoptosis) at the time of slice culture preparation. We, in alignment with Krassiokov et al., observe a relative tissue stabilization at 7 days after initial slice preparation, but are well aware that the preparation per se resulted in denervation with some glial activation (as presented by the GFAP, CD11b flow cytometry) within the cultures.

Immunohistochemistry with antibodies to nestin, DCX, MAP-2, NF, 5-HT, and TH revealed that the neuronal populations that are characteristic of first trimester development were maintained in the human slices.

In the present study, GFAP immunoreactivity was most apparent in the upper and lower periphery of slices. An increase in both signal intensity and enlarged GFAP^+^ cell bodies were revealed by applying immunohistochemistry on the slices. GFAP, the intermediate filament, is widely used as a marker for astrocytes. However, there are also other cells expressing GFAP such as progenitors which are not yet determined as astrocytes [52].

Both flow cytometric data and immunohistochemistry indicated an activated microglial response in the human slice cultures. Microglial cells are of mesodermal origin and initiate migration to the CNS during the first trimester embryonic stage, approximately 5–8 weeks postconception in human spinal cord [32, 53, 54], which is just the time interval we have studied.

Here we detected CD11b^+^CD45_low_ cell populations to observe overall microglial cells and HLA-DR/CD11b^+^CD45_low_ to identify activated microglial population, which has been described and applied by others [54]. CD68 is expressed highly in both macrophage and activated microglia but low in resting microglia. In order to further differentiate macrophage and activated microglia, we evaluated also CD11b CD45. Within the CNS, the CD11b^+^CD45_low_ cell population represents CNS microglia, in contrast to CD11b^+^CD45^high^ that is believed to represent the peripherally derived macrophage population [55, 56]. Resting microglia with ramified morphology also seldomly express MHC class II/ HLA-DR. Instead, HLA-DR expression is an early indicator of activation. We observed a significant increase in the HLA-DR/CD11b^+^CD45_low_ macrophage and monocyte populations in the SC slices, but not in the BS-SC slices, over time in culture. This might be due to the earlier migration of the mesodermal originating microglial cells into the human spinal cord compared to the brain stem during this developmental stage [32, 57].

Tissue slice culture allows maintenance and development of cellular networks in a microenvironment resembling in vivo condition more closely than cell culture, but with a risk that the structural and functional integrity might be lost over time in culture. In the present study, the calcium signaling and electrophysiological assessment confirmed that a good proportion of the human immature cells remained viable and presented spontaneous and evoked activity during culture, similar to what would be expected in an acutely processed spinal cord slice.

The calcium signaling analysis in the present study, revealed cells exhibiting spontaneous Ca^2+^ activity and ATP-induced Ca^2+^ elevations at 7–21 DIV with no significant difference between the two time points. It is tentative to think that most of the Ca^2+^ response observed post ATP stimulation was due to the neuronal cells that presented spontaneous calcium activity. However, immature neurons (or neuronal progenitors), radial glia, and astrocytes can all exhibit spontaneous Ca^2+^ activity [58]. Non-excitable cells such as microglia or endothelial cells can respond to ATP by elevating their cytosolic Ca^2+^ levels [59]. Therefore, the functional implications of these Ca^2+^ transients were not elucidated. Future studies aimed at deciphering the role of these Ca^2+^ responses might benefit using complementary markers, such as sulforhodamine 101, that can help distinguish different cell populations.

For the electrophysiology results, stimulation of the hOCs evoked a robust response with similar thresholds and magnitudes in all slices tested, clearly indicating well-preserved and homogeneous neuronal excitability along all hOCs. Moreover, the evoked response was reversibly blocked by TTX confirming an action-potential dependent neuronal transmission. Even though to our knowledge, the response to electrical stimulation has not been characterized in similar human ex vivo CNS slices preparation, the stimulation protocol and the responses recorded in this study are similar to what has been observed in other spinal cord preparations [60–62].

Both contusion injury and experimental human allogeneic neural cell therapy were applied in the hOCs. The experimental contusion injury significantly increased microglial activation and apoptosis. After grafting hfNPCs to the contusion injured slices, the degree of microglial activation and apoptosis no longer presented a significant difference compared to the sham control group. These findings are in line with SCI rodent models reporting anti-inflammatory [58–62] and neuroprotective effects by in vivo experimental cell therapy [63]. Overall, the human spinal cord slice culture injury model presented similarities of that in in vivo SCI animal models, i.e., loss of neural cells with increased apoptosis, astrocytic, and microglial reaction after contusion injury. However, there are also some clear drawbacks of the slice culture model compared to the in vivo situation, such as lack of an intact blood and lymphatic circulation, no possibility to follow complex functional locomotor behavior and to monitor potential adverse events such as allodynia. Studying in vitro “SCI” in the immature spinal cord, of course also limits the conclusions one may draw to human adult SCI conditions. However, the possibility to perform human study is limited, and adult postmortem tissues are rare with low survival and possibility to follow long term. Therefore, experimental human studies in the present hOC model system based on first trimester stages offer opportunity to additional gain of knowledge to narrow the gap between human and rodent model systems.

Experimental neural stem/progenitor cell therapy has resulted in beneficial effects including a reduction of the post-SCI inflammatory and glial response and host neural cell death in in vivo animal models, as reported by us and others [63–67]. In our previous study, transplanting equivalent donor human spinal cord-derived hfNPCs up to 9 days after SCI [63] resulted in improvement of function (as assessed by the BBB locomotor test) in two biomechanically different animal SCI models. The improvement correlated with increased host rodent spinal cord neuronal survival, indicating a neuroprotective mechanism. The vast majority of donor hfNPCs differentiated into astrocytes months after transplantation with no signs of mechanical allodynia. The therapeutic effect was not significant when a delayed transplantation was applied, again suggesting neuroprotection as the major mechanism of grafted hfNPCs. Cell type(s) offering a protective effect most likely was/were part of the immature NPCs present during the early observed 7–9 days of therapeutic window, since differentiation occurred multiple weeks to months after grafting. The ratios of various differentiated cell types were furthermore not correlated to functional improvement [63]. Therefore, in the present study of the human slice SCI model, we thoroughly evaluated the “host slice” apoptotic and glial cell population rather than the donor cell differentiation potential within this 14 days postgrafting time frame in the slices. Here, we confirm apoptotic and inflammatory characteristics of SCI as well as the potential of experimental cell therapy (previously observed in animal models) in human “SCI” using ex vivo slice cultures with allogeneic cell therapy. This model both allows and calls for further studies on more long-term allogeneic donor cell differentiation and interaction with human spinal cord slices as well as mechanism(s) behind the beneficial effects of human experimental neural cell therapy.

## Conclusions

In conclusion, we have further developed the human organotypic spinal cord slice culture technique and reported that longitudinal and cross-sectional human tissue slices can be cultured up to 21 DIV with remarkably consistent organotypic structural features and with confirmed viability by Ca^2+^ signaling and electrophysiological analysis. To experimentally challenge and apply the model, we performed in vitro SC slice contusion lesions and tested human allogeneic neural cell therapy with promising results. We conclude that human spinal cord slice cultures offer a suitable platform, which allows detailed studies of human CNS development and the evaluation of novel treatment strategies for CNS lesion/“SCI” in vitro with reduced species-specific obstacles. A reliable human CNS slice culture model may narrow the gap between cell suspension culture, in vivo animal models, and human clinical conditions. The human organotypic slice culture is flexible enough for advanced experimental procedures allowing us to test novel treatment strategies in a human-specific setting in parallel to cell culture and in vivo animal models prior to clinical application.

## Supplementary information

**Additional file 2 **: **Supplementary Table 1**. Antibodies used in immunohistochemistry. **Supplementary Table 2:** Antibodies used in flow cytometry. Information of the antibodies used in immunohistochemistry and flow cytometry.

**Additional files 3 **: **Supplementary Figure 1**. Phase contrast images for hOC. (A) A Representative BS-SC longitudinal slice deriving from 6-8.5w. and (B) 9-10.5 w., both at 21 DIV. In cases <8.5w., tissue architecture of BS and SC were preserved over time in culture. However, in older cases (>8.5w.) BS partly lost tissue integrity and tissue thickness was reduced to few cell layers, edges were uneven, and dark spots disappeared from BS as seen in (B). Arrows: dark spots; arrowheads: dark aggregates. (C) A representative SC cross sectional slice at 21 DIV, deriving from 7.5-8w. Arrows: dark spots; arrowhead: dark aggregates. (D) A SC slice from 5.5w. at 21 DIV. Tissue maintained its appearance but at this stage studied landmarks were not fully developed. (E) SC slice deriving from 6w at 21 DIV. Fibers appeared from 7 DIV and they grew creating a network in culture as seen in (D, E). (F) In vitro phase contrast micrograph and (G) Cresyl violet stained hOC SC slices cultured under serum and glucose deprivation for one week. The in vitro slices lost tissue integrity, edges were uneven and was becoming very thin. Bar=0.6 mm. Abbreviations: PO, pons; MO, medulla oblongata; SC, spinal cord; WM: white matter; DF, dorsal funiculi and or dorsal septum; VF, ventral median fissure and or ventral funiculi; DH, dorsal horns or alar plate; VH, ventral horns or basal plate; CC, central canal and or extra canalicula. **Supplementary Figure 2**. Flow cytometric quantification of proliferation, apoptosis, glial cells, microglia on BS-SC and SC slices. Flow cytometric quantification of proliferation (A, B), apoptosis (C, D) and GFAP expression (E, F) and CD11b^+^CD45_low_ expressing cells (G, H) in BS-SC (A, C, E, G) and SC (B, D, F, H) slices cultures, grouped depending on original weeks post conception. (A, B) proliferation increased significantly from 7DIV to that after 21 DIV in slices derived from 5-6.5w. in both BS-SC (A; p<0.01) and SC (B; p<0.05) slice cultures. At 21 DIV, BS-SC slices derived from 5-6.5w. presented double the percentage of proliferating cells compared to that at 9-10.5w. (A; p<0.05). (C, D) In the slices, the amount of apoptotic cells was relatively stable during cultures from 7DIV to 21 DIV, while the percentages of caspase-3^+^ cells at 14 and 21 DIV were often significantly higher compared to that in situ (p<0.05). At 7 DIV the proportion of apoptotic cells was higher in 9-10.5w. compared to 5-6.5w. (p<0.05). (E, F) No significant differences were detected by flow cytometry in the percentage of GFAP^+^ cells among groups at same DIV or over time. Values are presented as mean ± SEM. *p<0.05; **p<0.01. **Supplementary Figure 3**. Immunostaining of proliferating and apoptotic cells in BS-SC and SC slices. (A-L) Representative images of Ki-67 (red), caspase-3 (green) and DAPI (blue) immunofluorescent staining on SC (A, C, D, G, H and K) and BS-SC (B, E, F, I, J and L) slices of different time points (in situ, 7 DIV, 14 DIV and 21 DIV). For the in situ and 21 DIV images of Ki-67, please see Fig. 1. **Supplementary Figure 4**. HLA-DR quantification and representative dot plots of the flow cytometric analysis. (A-B) Representative images of HLA-DR immunofluorescent staining of BS-SC slices of 7 DIV (A) and 14 DIV (B). (C) Quantification of HLA-DR^+^ cells. The image analysis was based on BS-BC slices 7 DIV and 14 DIV (3-4 sections per condition). Images were randomly taken in both conditions. DAPI^+^ cells were counted automatically by ImageJ, with the same filter setting for all sections. HLA-DR^+^DAPI^+^ cells were considered as HLA-DR^+^ cells. Values are presented as mean ± SEM. Bars=0.1mm. (D-E) Representative dot plots of the flow cytometric analysis of glial cell populations. (F) Representative dot plots over the hematopoietic cell populations, macrophages and monocytes. Gating is set from the negative isotype controls. Gating strategy: (Da, Db) microglia, CD11b^+^/ CD45_low_; (Da, Db, Dc) activated microglia, CD11b^+^/CD45_low_/HLA-DR^+^; (Fa, Fb) macrophages, CD11b^+^/CD45^high^; (Fa, Fb) monocytes, CD11b-/CD45^+^ cells. Abbreviations: Iso, mouse IgG isotype control for the respective fluorochromes. **Supplementary Figure 5**: Phase contrast images of contusion/cut SCI with hfNPC grafts. Data description please see respective figure legends. (A-I) “Donor” allogeneic hfNPCs grafted to “host” slices (G-I) subjected to contusion SCI and compared to contusion SCI alone (D-F) or to sham control slices (A-C). (J-W) GFP-hfNPC graft in spinal cord slices. After culture for 9DIV, a mechanical cut in the dorso-lateral spinal cord was performed. 1 hour later, GFP-hfNPCs were grafted at the injury site. Images taken with phase contrast and/or fluorescence microscope at 1h, 3 or 7 days postinjury. J-M) sham control; N-Q) cut SCI; R-U) cut SCI+ GFP-hfNPCs. V and W) represent green GFP-hfNPC grafted in the slices imaged in T and U with increased magnification. Bar: a-l) 1mm; m-n) 0.5mm. Size of slices is presented in mm. Score represents the total hOC Score.

## Data Availability

All data generated or analyzed during this study are included in this published article and its supplementary information files.

## Abbreviations

aCSF: Artificial cerebrospinal fluid
BS-SC: Brain stem-spinal cord
CNS: Central nervous system
DIV: Days in vitro
DPI: Days postinjury
hOC: Human organotypic culture
hfNPC: Human first trimester-derived neural stem/progenitor cell
PBS: Phosphate-buffered saline
PFA: Paraformaldehyde
PO: Pontine
RT: Room temperature
SC: Spinal cord
SCI: Spinal cord injury
TTX: Tetrodotoxin
w.: Weeks postconception
