# Correction to: Human ex vivo spinal cord slice culture as a useful model of neural development, lesion, and allogeneic neural cell therapy

**DOI:** 10.1186/s13287-020-01893-3

**Published:** 2020-08-27

**Authors:** Chenhong Lin, Cinzia Calzarossa, Teresa Fernandez-Zafra, Jia Liu, Xiaofei Li, Åsa Ekblad-Nordberg, Erika Vazquez-Juarez, Simone Codeluppi, Lena Holmberg, Maria Lindskog, Per Uhlén, Elisabet Åkesson

**Affiliations:** 1 Department of Neurobiology, Care Sciences and Society, Div. of Neurogeriatrics, Karolinska Institutet, Stockholm, Sweden; 2 Department of Neurology and Laboratory of Neuroscience, Università degli Studi diMilan, Milan, Italy; 3 Division of Molecular Neurobiology, Departmentof Medical Biochemistry and Biophysics, Karolinska Institutet, Stockholm, Sweden; 4 Department of Neurology, Shengjing Hospital of China Medical University, Shenyang, People’s Republic of China; 5 Department of Clinical Science, Intervention and Technology, Div. of Obstetrics and Gynecology, Karolinska Institutet, Stockholm, Sweden; 6 The R&D Unit, Stockholms Sjukhem, Stockholm, Sweden

**Correction to: Stem Cell Res Ther 11, 320 (2020)**

**https://doi.org/10.1186/s13287-020-01771-y**

Following publication of the original article [1], the authors would like to add below sentence under the heading **Acknowledgement.**

We also want to acknowledge that this study was partially performed at the LCI facility/Nikon Center of Excellence, Karolinska Institutet, supported by grants from the Knut and Alice Wallenberg Foundation, Swedish Research Council, KI infrastructure, Centre for Innovative Medicine and Jonasson center at the Royal Institute of Technology.
