# Supplementary material for: Human ex vivo spinal cord slice culture as a useful model of neural development, lesion, and allogeneic neural cell therapy
Source: Stem Cell Res Ther. 2020 Jul 29;11:320. doi: 10.1186/s13287-020-01771-y (PMC7390865; doi:10.1186/s13287-020-01771-y)
Supplement: Supplementary file 2 — Additional file 2 : Supplementary Table 1. Antibodies used in immunohistochemistry. Supplementary Table 2: Antibodies used in flow cytometry. Information of the antibodies used in immunohistochemistry and flow cytometry. [file 13287_2020_1771_MOESM2_ESM.docx]

**Supplementary table 1: Antibodies used in immunohistochemistry**

| **Antibody** | **Species** | **Concentration** | **Suppliers** |
| --- | --- | --- | --- |
| ***Primary Antibodies:*** |  |  |  |
| Ki67 | Mouse | 1:100 | DAKO |
| Nestin | Rabbit | 1:400 | Merck-Millipore |
| Nestin | Mouse | 1:100 | Merck-Millipore |
| Doublecortin (DCX) | Rabbit | 1:500 | ABCAM |
| DCX | Guinea pig | 1:1000 | Merck-Millipore |
| Microtubular associated protein 2 (MAP-2) | Rabbit | 1:200 | Merck-Millipore |
| MAP-2 | Mouse | 1:100 | Merck-Millipore |
| Neurofilament (NF), 70KDa | Mouse | 1:200 | Immunotech |
| Tyrosine Hydroxylase (TH) | Mouse | 1:1000 | Merck-Millipore |
| 5-hydroxytripamine (5HT) | Rabbit | 1:500 | Immunostar |
| Glial fibrillary acidic protein (GFAP) | Mouse | 1:200 | Merck-Millipore |
| Ionized calcium-binding adapter molecule 1 (Iba 1) | Rabbit | 1:200 | WAKO |
| Laminin α1 | Mouse | 1:200 | Lifespan Bioscience |
| Human Leukocyte Antigen-DR isotype (HLA-DR) | Mouse | 1:50 | DAKO |
| Cleaved Caspase 3 | Rabbit | 1:100 | Cell Signaling Technology |
|  |  |  |  |
| ***Secondary Antibodies:*** |  |  |  |
| Anti-rabbit AlexaFluo488 | Goat | 1:1600 | Merck-Millipore |
| Anti-mouse AlexaFluo488 | Goat | 1:1200 | Merck-Millipore |
| Anti-guinea pig AlexaFluo488 | Goat | 1:1200 | Merck-Millipore |
| Anti-rabbit Cy3 | Goat | 1:2000 | Jackson’s Laboratories |
| Anti-mouse Cy3 | Goat | 1:2000 | Jackson’s Laboratories |
|  |  |  |  |

**Supplementary table 2: Antibodies used in flow cytometry**

| **Antibodies** | **Fluorescence conjugated** | **Clone** |
| --- | --- | --- |
| Ki67 | PE-Cy7 | B56, IgG1 |
| Active Caspase 3 | PE | C92-605, IgG |
| GFAP | AlexaFluor-647 | 1B4, IgG2b |
| CD11b | PE-Cy7 | M1/70, IgG2b |
| CD45 | APC | HI30, IgG1 |
| HLA-DR | PE | G46-6, IgG2a |
| CD68 | AlexaFluor-647 | Y1/82A, IgG2b |
|  |  |  |

Note: all antibodies in this table are from BD Bioscience
