# Supplementary material for: Human ex vivo spinal cord slice culture as a useful model of neural development, lesion, and allogeneic neural cell therapy
Source: Stem Cell Res Ther. 2020 Jul 29;11:320. doi: 10.1186/s13287-020-01771-y (PMC7390865; doi:10.1186/s13287-020-01771-y)

Supplementary figure 1:

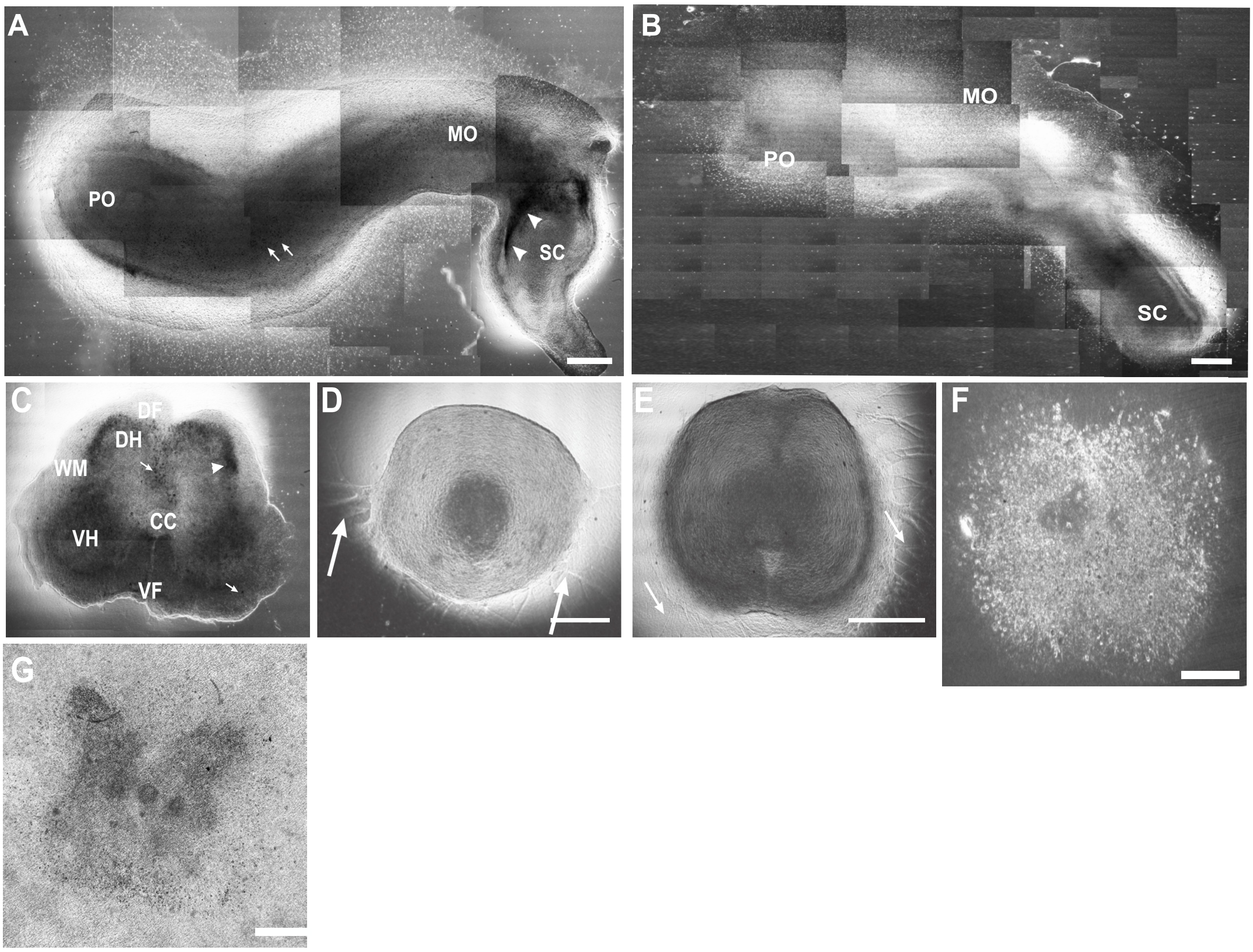

Supplementary figure 2:

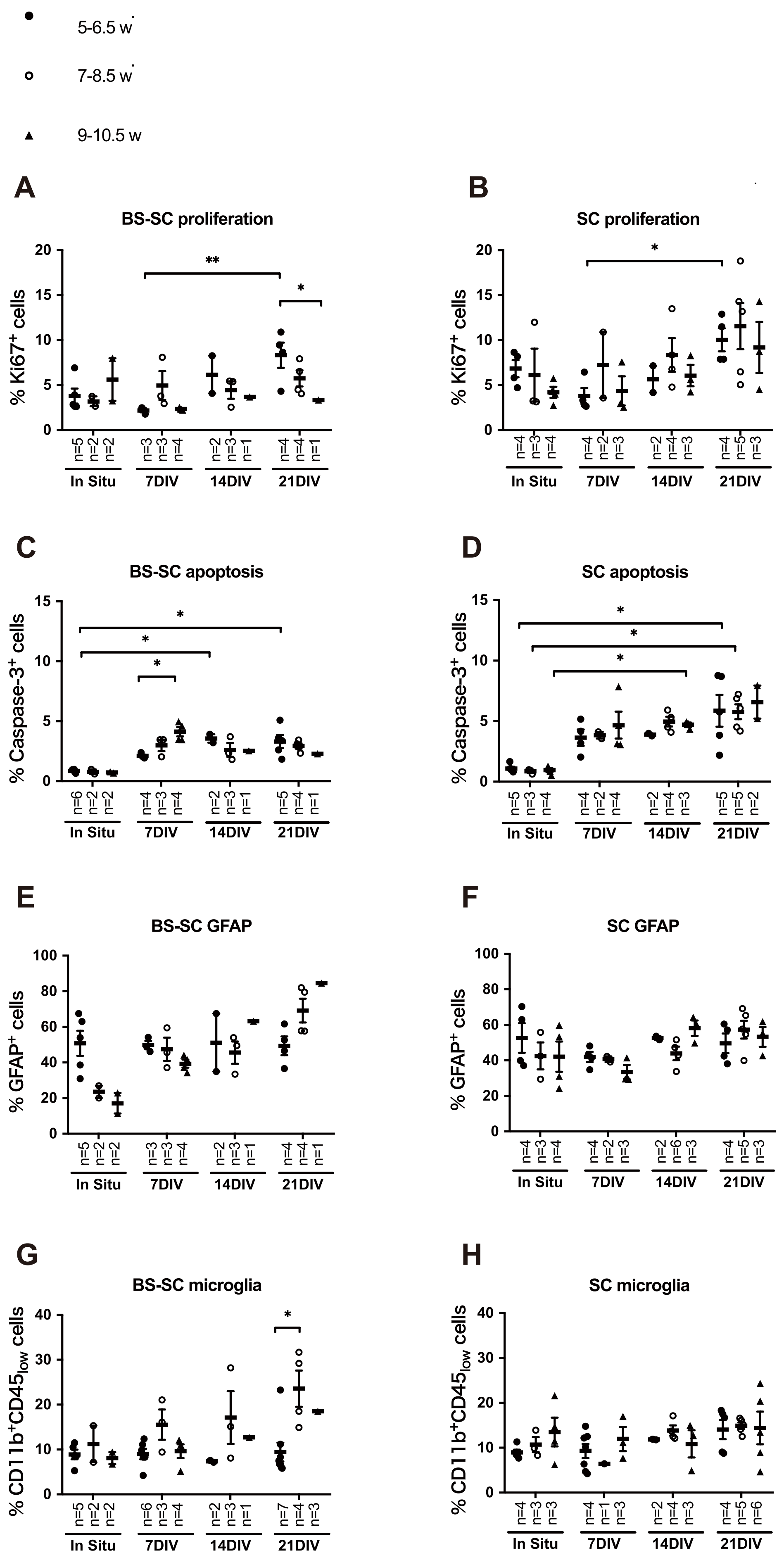

Supplementary figure 3:

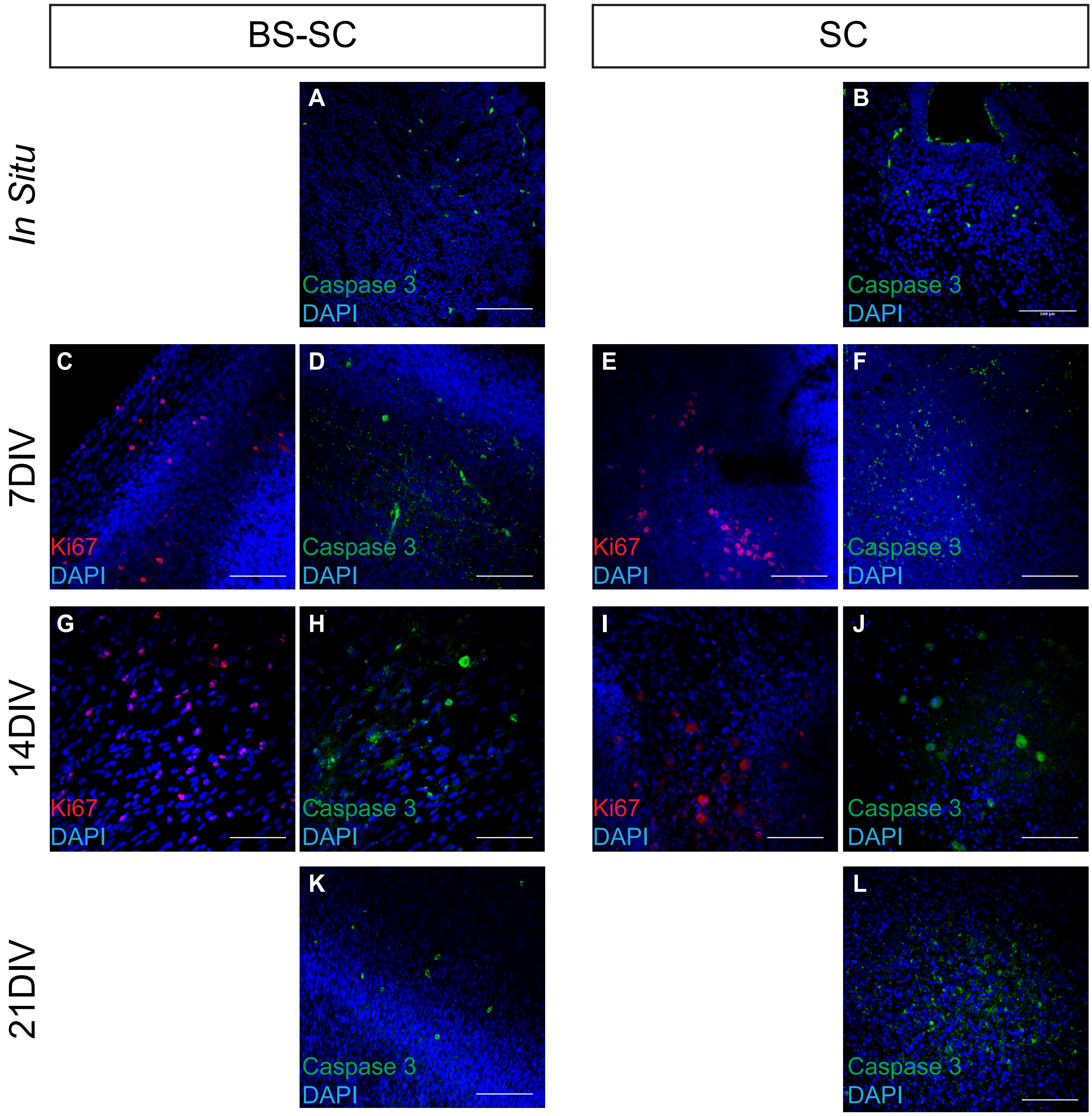

Supplementary figure 4:

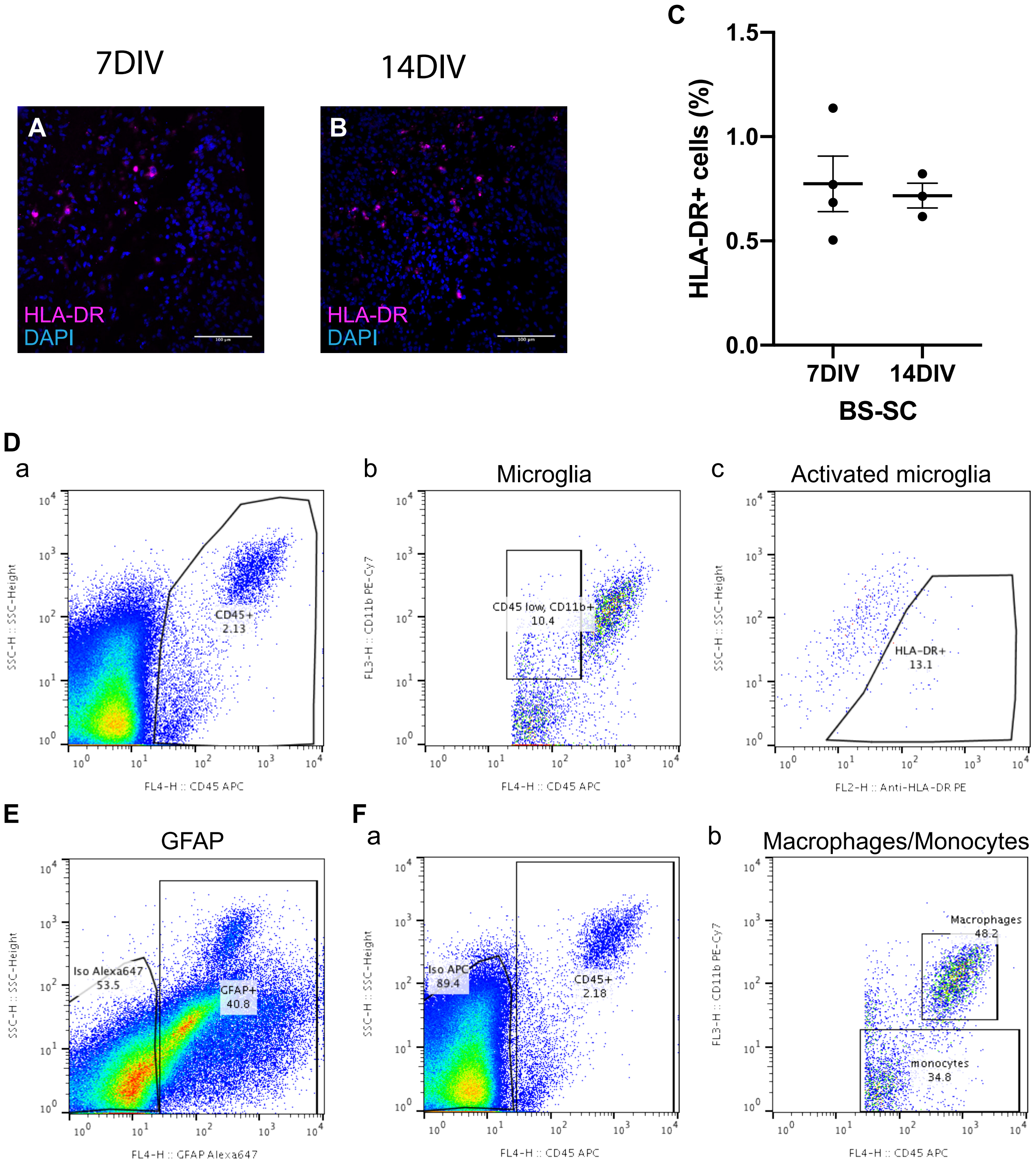

Supplementary figure 5:

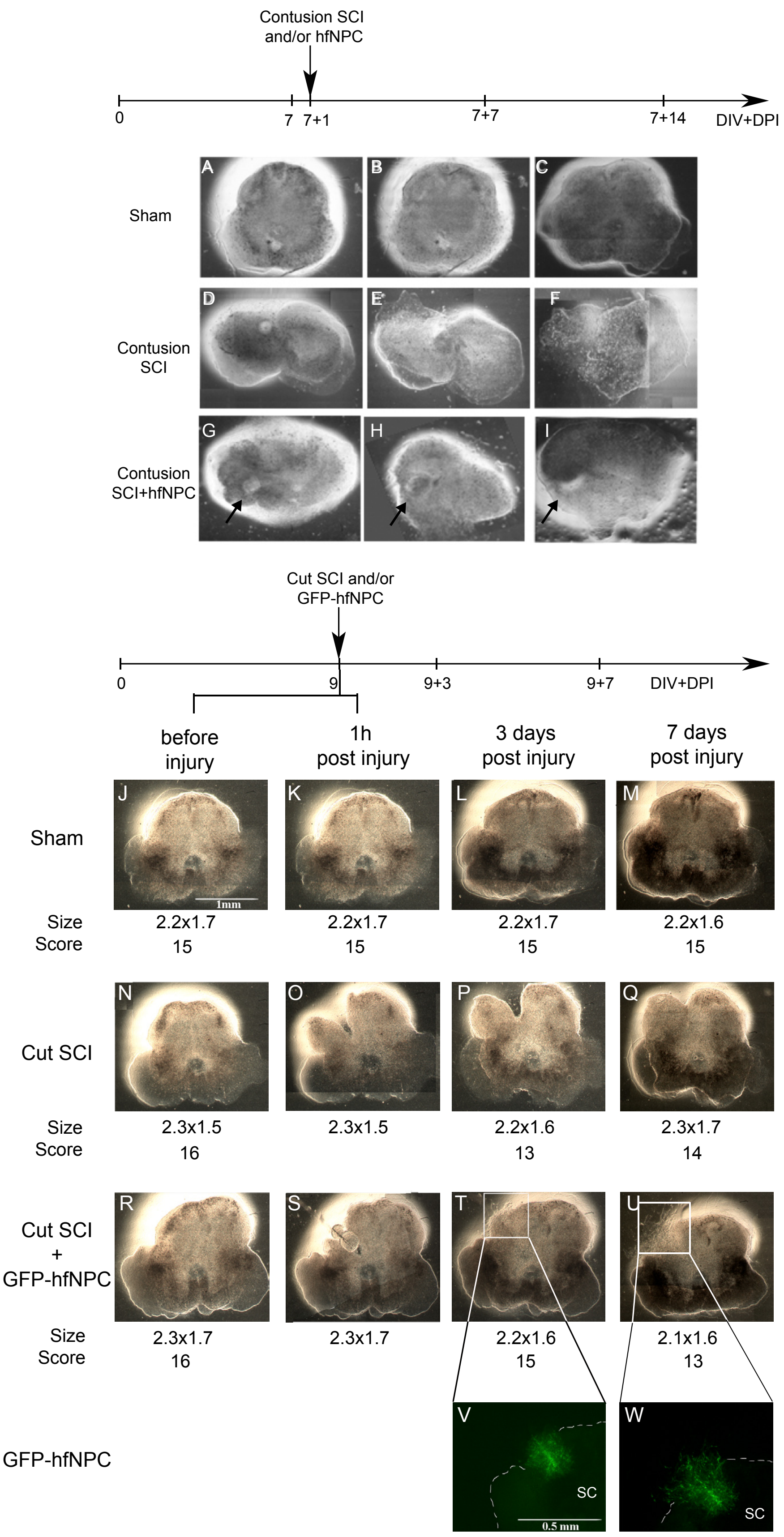

Supplement: Supplementary file 3 — Additional files 3 : Supplementary Figure 1. Phase contrast images for hOC. (A) A Representative BS-SC longitudinal slice deriving from 6-8.5w. and (B) 9-10.5 w., both at 21 DIV. In cases <8.5w., tissue architecture of BS and SC were preserved over time in culture. However, in older cases (>8.5w.) BS partly lost tissue integrity and tissue thickness was reduced to few cell layers, edges were uneven, and dark spots disappeared from BS as seen in (B). Arrows: dark spots; arrowheads: dark aggregates. (C) A representative SC cross sectional slice at 21 DIV, deriving from 7.5-8w. Arrows: dark spots; arrowhead: dark aggregates. (D) A SC slice from 5.5w. at 21 DIV. Tissue maintained its appearance but at this stage studied landmarks were not fully developed. (E) SC slice deriving from 6w at 21 DIV. Fibers appeared from 7 DIV and they grew creating a network in culture as seen in (D, E). (F) In vitro phase contrast micrograph and (G) Cresyl violet stained hOC SC slices cultured under serum and glucose deprivation for one week. The in vitro slices lost tissue integrity, edges were uneven and was becoming very thin. Bar=0.6 mm. Abbreviations: PO, pons; MO, medulla oblongata; SC, spinal cord; WM: white matter; DF, dorsal funiculi and or dorsal septum; VF, ventral median fissure and or ventral funiculi; DH, dorsal horns or alar plate; VH, ventral horns or basal plate; CC, central canal and or extra canalicula. Supplementary Figure 2. Flow cytometric quantification of proliferation, apoptosis, glial cells, microglia on BS-SC and SC slices. Flow cytometric quantification of proliferation (A, B), apoptosis (C, D) and GFAP expression (E, F) and CD11b+CD45low expressing cells (G, H) in BS-SC (A, C, E, G) and SC (B, D, F, H) slices cultures, grouped depending on original weeks post conception. (A, B) proliferation increased significantly from 7DIV to that after 21 DIV in slices derived from 5-6.5w. in both BS-SC (A; p<0.01) and SC (B; p<0.05) slice cultures. At 21 DIV, BS-SC slices d [file 13287_2020_1771_MOESM3_ESM.pdf]
